# Computed Tomography-Based Radiomics Model to Preoperatively Predict Microsatellite Instability Status in Colorectal Cancer: A Multicenter Study

**DOI:** 10.3389/fonc.2021.666786

**Published:** 2021-07-01

**Authors:** Zhi Li, Qi Zhong, Liang Zhang, Minhong Wang, Wenbo Xiao, Feng Cui, Fang Yu, Chencui Huang, Zhan Feng

**Affiliations:** ^1^ Department of Radiology, the First Affiliated Hospital, Zhejiang University School of Medicine, Hangzhou, China; ^2^ Department of Radiology, Hangzhou Hospital of Traditional Chinese Medicine, Hangzhou, China; ^3^ Department of Radiology, Cancer Hospital of the University of Chinese Academy of Sciences (Zhejiang Cancer Hospital), Hangzhou, China; ^4^ Department of Radiology, First Affiliated Hospital of Wannan Medical College, Wuhu, China; ^5^ Department of Pathology, The First Affiliated Hospital, Zhejiang University School of Medicine, Hangzhou, China; ^6^ Department of Research Collaboration, R&D Center, Beijing Deepwise & League of PHD Technology Co, Ltd, Beijing, China

**Keywords:** computed tomography, logistic regression, microsatellite instability, colorectal cancer, AUC

## Abstract

**Objectives:**

To establish and validate a combined radiomics model based on radiomics features and clinical characteristics, and to predict microsatellite instability (MSI) status in colorectal cancer (CRC) patients preoperatively.

**Methods:**

A total of 368 patients from four hospitals, who underwent preoperative contrast-enhanced CT examination, were included in this study. The data of 226 patients from a single hospital were used as the training dataset. The data of 142 patients from the other three hospitals were used as an independent validation dataset. The regions of interest were drawn on the portal venous phase of contrast-enhanced CT images. The filtered radiomics features and clinical characteristics were combined. A total of 15 different discrimination models were constructed based on a feature selection strategy from a pool of 3 feature selection methods and a classifier from a pool of 5 classification algorithms. The generalization capability of each model was evaluated in an external validation set. The model with high area under the curve (AUC) value from the training set and without a significant decrease in the external validation set was final selected. The Brier score (BS) was used to quantify overall performance of the selected model.

**Results:**

The logistic regression model using the mutual information (MI) dimensionality reduction method was final selected with an AUC value of 0.79 for the training set and 0.73 for the external validation set to predicting MSI. The BS value of the model was 0.12 in the training set and 0.19 in the validation set.

**Conclusion:**

The established combined radiomics model has the potential to predict MSI status in CRC patients preoperatively.

## Introduction

Colorectal cancer (CRC) arises from the mucous lining of the colon or rectum, and is one of the most common forms of digestive system cancer. According to 2018 statistics (1), the worldwide incidence and mortality of CRC both ranked 6th among all cancers in males, while incidence and mortality ranked 4th and 5th, respectively, in females. Up to 12–15% of all CRC patients have a peculiar molecular phenotype: microsatellite instability (MSI) (2). MSI is the result of DNA mismatch repair (MMR) protein defects, and also renders a high mutation burden to the genome, increasing the risk of tumorigenesis. The MSI phenotype was first reported in CRC in 1993 (3). Based on MSI status, patients can be classified into three groups: high-frequency MSI (MSI-H), low-frequency MSI (MSI-L), and MSI-stability (MSS). Currently, MSI testing is recommended by the colorectal cancer diagnosis and treatment guidelines for all CRC patients.

MSI-H cancer has many distinct characteristics compared with MSI-L and MSS. First of all, MSI-H is a predictive factor for the adjuvant chemotherapy response. In addition, patients with MSI-H CRC stage II usually have poorly differentiated tumors, but a better prognosis. It is not recommended that these patients receive 5-FU-based adjuvant chemotherapy treatment (4–6). Moreover, MSI-H is a predictor for immunotherapy efficacy in late-stage solid tumors. In 2016, the European Society of Medical Oncology (ESMO) consensus guidelines stated that MSI testing had strong predictive value for the efficacy of immune checkpoint inhibitors in treating patients with metastatic CRC.

Currently, MSI testing is performed postoperatively using the surgically collected specimen, including immunohistochemistry (IHC) and DNA-based assays. Both of these are the most desirable methods. However, in some cases, for example, in patients who have developed metastases, additional invasive procedures are not necessary, in addition, when a patient received DNA-based MSI assays, but the test failed due to compromised DNA quality (whether because of the small biopsy sample or poor fixation technique). The patient had to be biopsied again. In this case, the use of non-invasive methods to predict MSI status is valuable.

Radiomics intuitively and quantitatively describes the morphological features of the tumor using radiological images, and has strong predictive value for cancer prognosis, treatment guidance and treatment efficacy evaluation (7, 8). Cancer intrinsic molecular subtypes can produce heterogeneity, which can be predicted by the radiomics prediction model in a non-invasive and convenient way without asking patients to undergo unnecessary tests. Currently, radiomics has been extensively investigated in the cancer research field, and has demonstrated some clinical value (9–13). However, radiomics has been infrequently used to predict MSI status in CRC patients. The preliminary studies by Fan et al. (14) and Pernicka et al. (15) showed the potential of radiomics based on contrast-enhanced CT images to predict MSI status. However, their studies come from a single-center, and were not externally validated. MSI status determines the therapeutic regimen of CRC. Therefore, identifying a radiological biomarker that could predict MSI status is of great significance for precise CRC management. This study aimed to construct a radiomics model using both clinical and radiological characteristics, and subsequently to use external data from multiple institutions to evaluate the model’s generalization ability for preoperative prediction of MSI status in CRC patients.

## Materials and Methods

### Patients

This retrospective study was approved by the Institutional Review Board of the First Affiliated Hospital of Zhejiang University School of Medicine, the First Affiliated Hospital of Wannan Medical College, Cancer Hospital of the University of Chinese Academy of Sciences and Hangzhou Hospital of Traditional Chinese Medicine. The signed informed consent forms were waived. This study was conducted in accordance with the Declaration of Helsinki. Patients who were diagnosed CRC and treated in the four participating institutions between January 2017 and September 2019 were enrolled in the study. The inclusion criteria were as follows. (1) Pathology reports with diagnosis of CRC; (2) Patient received abdominal contrast-enhanced CT examination within one week before surgery; (3) Pathology report indicated MSI status as assessed by IHC staining. The exclusion criteria were: (1) any type of anti-cancer treatments, such as radiation therapy, chemotherapy or biological therapy before the CT examination; (2) incomplete clinical data; (3) poor CT image quality (e.g. presence of artifacts); or (4) the lesion was too small and doesn’t appear clearly on the CT image.

The clinical data were retrospectively reviewed. The clinical characteristics, including age, gender, location of the tumor (right colon, left colon), CEA status (normal or abnormal), and CA199 status (normal or abnormal) were recorded.

### MSI Status Assessment

In this study, the MSI status was evaluated based on the expression levels of MMR gene protein products (MLH1, MSH2, MSH6, and PMS2), which were assessed by IHC staining. The IHC staining results were read and agreed by two pathologists with over ten years of experience in abdominal cancer diagnosis. The patients were divided into two groups: the MS-L/S group had positive staining of all four MMR proteins, while the MSI-H group included patients for which any one of the MMR proteins tested negative.

### CT Imaging Acquisition

CT images were acquired using six different CT scanners from four institutions. All patients received a preoperative abdominal contrast-enhanced CT scan. Contrast-enhanced CT examinations in Institution I were conducted using three CT scanners, including a 64-slice and a 256-slice CT scanner (Philips Healthcare), as well as a 16-slice CT scanner (Toshiba Medical System). In Institution II, the CT scans were performed using two CT scanners, including a 64-slice CT scanner (Siemens Healthineers) and a 16-slice CT scanner (Philips Healthcare). The CT scans in Institution III were undertaken using a 64-slice CT scanner (GE Healthcare). The CT scans in Institution IV were conducted using a 256-slice CT scanner (Philips Healthcare). Mean acquisition parameters in the four institutions were: tube voltage of 120 kev (100–130 kev), tube current of 213 mAs (125–300 mAs), pitch of 0.6 to 1.25 mm, slice thickness of 3 to 5 mm, and reconstruction interval of 3 to 5 mm. The contrast agents (Bayer Schering Pharma) were bolus-injected (1.5 mL/kg) at the rate of 2.5–3.5 ml/s with a high-pressure syringe. CT scans of the arterial phase and portal venous phase were carried out at 25 to 35 seconds and 55 to 75 seconds after injection, respectively.

### Tumor Segmentation and Feature Extraction

**Figure 1:**
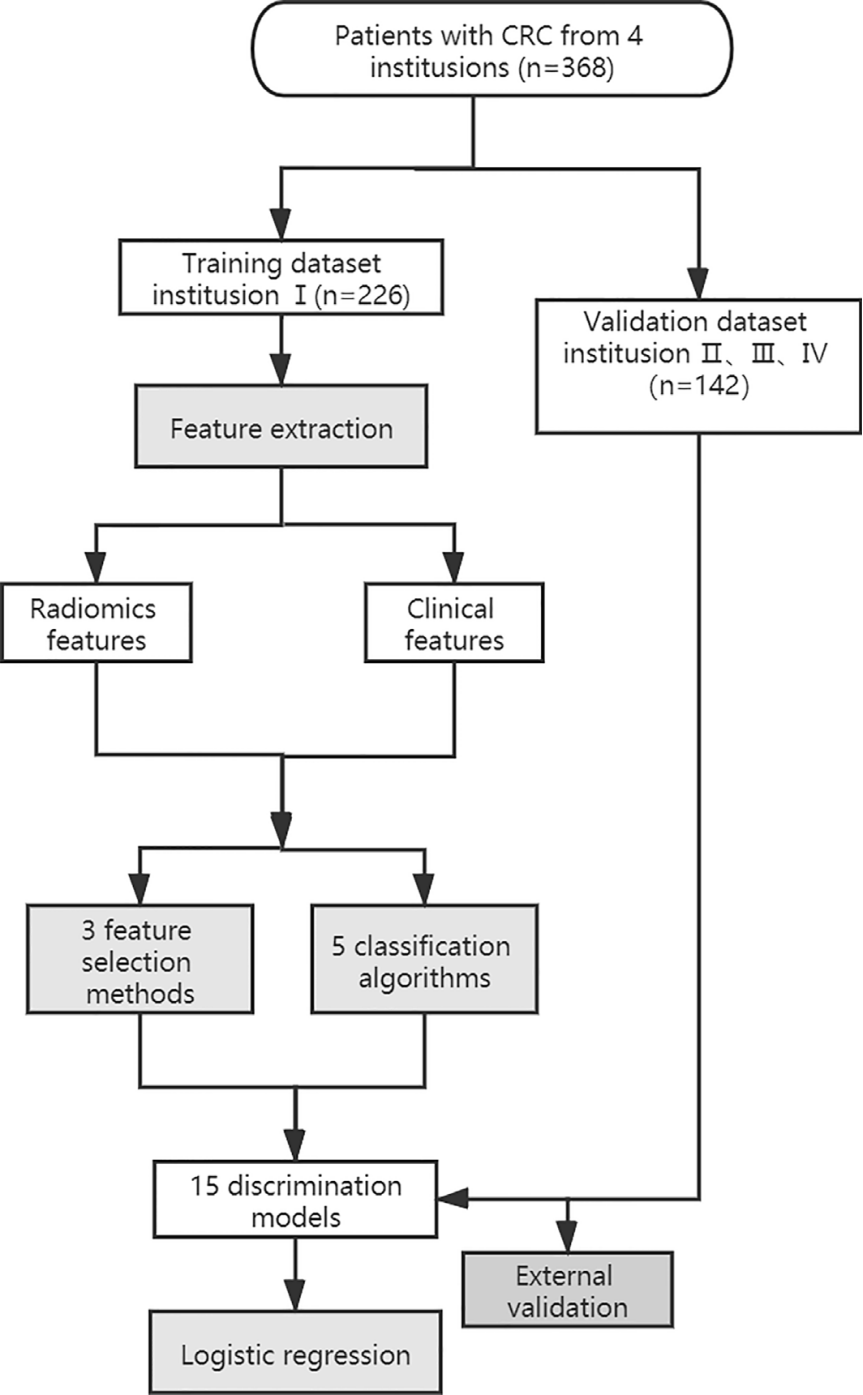
Schematic shows workflow for this study.

**Figure 2:**
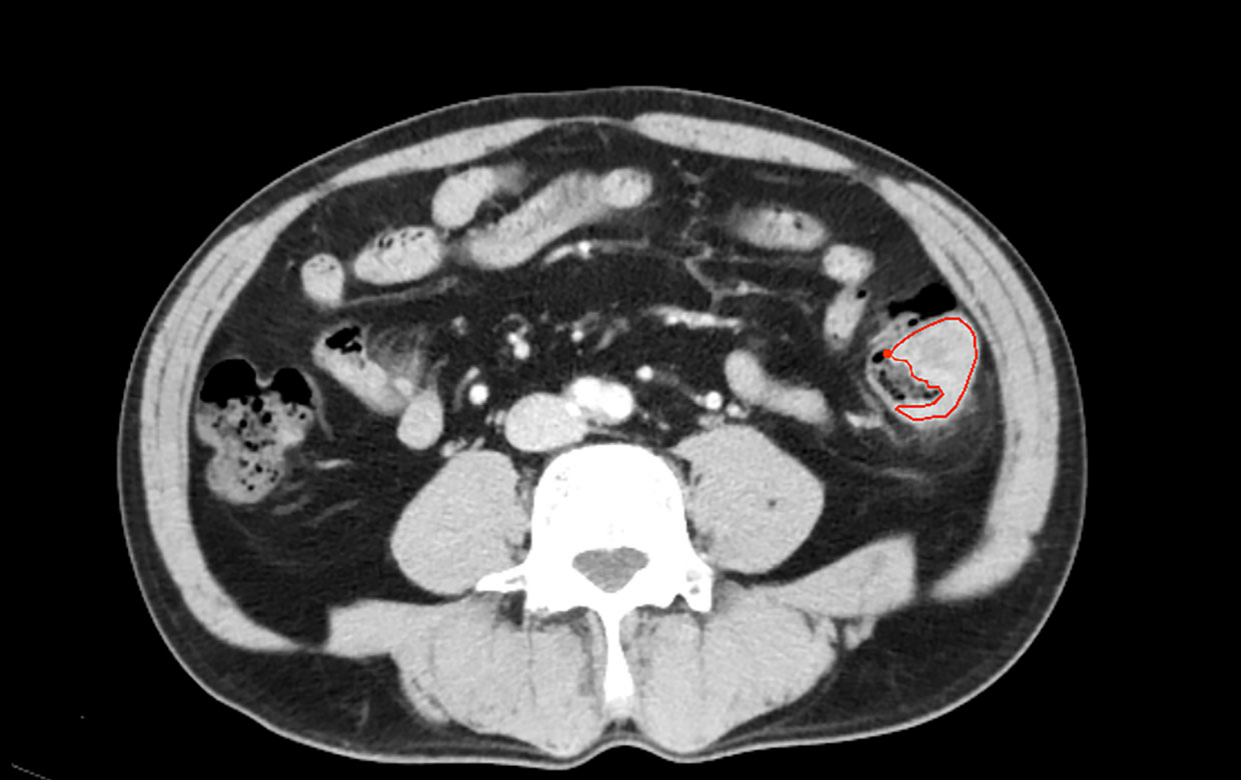
One patient with descending coloncancer, male, 52years old. The area inside the red line represents the ROI for the tumor.

The workflow of the radiomics analysis is shown in **Figure 1**. The tumor region was segmented manually by two experienced radiologists in the portal phase of enhancement using the IBEX software package created in Matlab (16). Both radiologists were blinded to the MSI status before ROI segmentation. Radiologist 1 (with 10 years of experience) performed the segmentation of all CRC tumors twice with a 3-months interval. Radiologist 2 (with 5 years of experience) performed the segmentation of all tumors once. Based on the CT images of portal venous phase, the two radiologists first selected and outlined the largest axial diameter of the tumor and then outlined adjacent lower and upper slices of the tumor. The region of interest (ROI) included the necrotic or hemorrhagic area within the tumor, but the normal colorectal wall, adjacent mesenteric fat tissue, and bowel contents were avoided as much as possible (**Figure 2**).

Before feature extraction, all the CT images were resampled to a voxel size of 1 × 1 × 1 mm and normalized to a 1–64 gray level. Radiomics features extracted from each ROI included Intensity Histogram (n = 49), Gray Level Co-occurrence Matrix (n = 1518), Gray Level Run Length Matrix (n = 33), Neighbor Intensity Difference (n = 10), and shape (n = 18). A total of 1628 features were extracted. The clinical characteristics included age, gender, tumor location, CA19-9 status, and CEA status.

### Dimensionality Reduction and Establishment of the Model

The intra-/inter-class correlation coefficients (ICCs) were used to estimate the variability between radiologist 1 and radiologist 2 in tumor segmentation. The stable features with ICCs of > 0.8 remained. Radiomics features were then subjected to spearman correlation analysis with a correlation coefficient threshold of 0.8. The categorical variables of the clinical characteristics were converted by applying one-hot encoding. The filtered radiomic features and clinical characteristics of statistical significance were combined. We built radiomics models based on a feature selection strategy from a pool of 3 feature selection methods(MI, L1- regularization, Tree model), as well as a classifier from a pool of 5 classification algorithms [Logistic regression, Support vector machine (SVM), Random forest, Gradient boosting machine (GBM), Naive Bayes], thus, resulting in a total of 15 different discrimination models (**Table 2**). We evaluated each of these models with 10-fold cross-validation, in each of which an optimal subset of features were first established by a specific feature selection method, and then the prescreened features were fed into a classifier for discrimination and modeling. The discriminative ability of the model was evaluated on the basis of receiver operating characteristic (ROC) curves and the area under the curve (AUC). Delong test was used to evaluate the AUC values of the model in the training set and validation set. The generalization capability of each model was evaluated in an external validation set. According to the AUC from the training set and the external validation set, a model with a higher AUC value from the training set and without a significant decrease in the external validation set was selected. Then, the BS was used to quantify overall performance of the selected model. BS is the mean squared difference between the observed and predicted outcome. It is a combination of calibration and differentiation. If the model performs perfectly overall and the predicted value is exactly the same as the actual value, then the BS is equal to 0, if the BS >0.25, the model is considered worthless.

### Statistical Analysis

The clinical data of the training dataset and validation dataset were analyzed using descriptive statistics. The numerical data (age) were compared using the *t* test, while the categorical data (gender, location of the tumor, CEA status, CA199 status) were compared using the chi-square test. All statistical analyses were performed using R software (version: 3.4.1; http://www.rproject.org).

## Results

Based on the inclusion and exclusion criteria, 368 patients from four institutions were included in the study. There were 186 MSI-H patients, with 115 from Institution I, 71 from Institution II、Institution III and Institution IV. There were 182 MS-L/S CRC patients, with 111 from Institution I, 71 from Institution II、Institution III and Institution IV. A total of 193 males and 175 females were included (average age: 56 years old; range: 23–92 years old). The 226 patients from Institution I were used as a training dataset for the design of the prediction model, while the 142 patients from the other three institutions were used as the validation dataset.

The clinical characteristics of the CRC patients were shown in **Table 1**. In both training and validation datasets, the patient age and tumor location differed significantly between MSI-H and MS-L/S groups (p<0.05), while gender, CA199, and CEA status were comparable between groups (p>0.05). The clinical characteristics did not differ significantly between the training dataset and the validation dataset (p>0.05).

**Table 1:** Characteristics of CRC patients in the MSI-H group and MS-L/S group.

Characteristics	Training set		*P*	Validation set		*P*	P*
	MSI-H (n=115)	MS-L/S (n=111)		MSI-H (n=71)	MS-L/S (n=71)		
Age (years, range)	23-82	28-92	<0.05	25-79	28-88	<0.05	0.067
Gender			0.900			0.736	0.663
Male	60	56		37	40		
Female	55	55		34	31		
Tumor location			<0.05			<0.05	0.368
right colon	88	41		50	26		
left colon	14	38		11	33		
CA19-9			0.252			0.169	0.174
Normal	77	83		50	58		
Abnormal	38	28		21	13		
CEA			0.097			0.139	0.507
Normal	71	81		46	55		
Abnormal	44	30		25	16		

P*, Statistic difference between the training dataset and the validation dataset.

**Figure 3:**
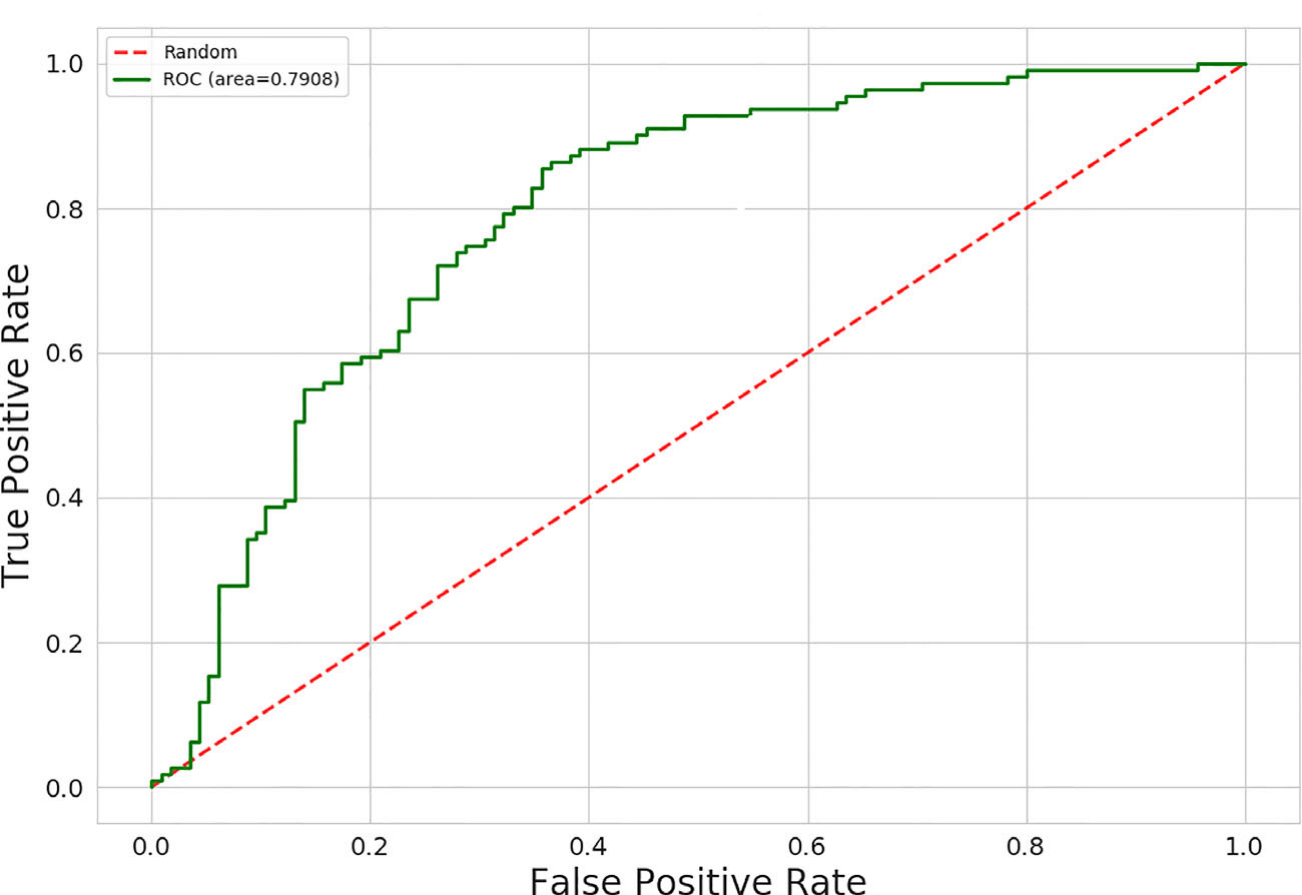
The ROC curves of the radiomics signature in the training set.

The AUC values for 15 different discrimination models were shown in **Table 2**. The logistic regression model using the MI dimensionality reduction method performed better in the training set with an AUC value of 0.79 [95% confidence intervals (CI): 0.73–0.85] (**Figure 2**) and was not significantly reduced (p=0.19) in the validation set with an AUC value of 0.73 (95%CI: 0.65-0.80) (**Figure 3**). The AUC value indicated that the established radiomics model could predict CRC MSI status preoperatively with satisfactory performance. The training dataset had an accuracy of 0.73, sensitivity of 0.77, and specificity of 0.68. The external validation dataset had an accuracy of 0.69, sensitivity of 0.67,and specificity of 0.72. The BS value of the model was 0.12 in the training set and 0.19 in the validation set.

**Table 2:** AUC value for different combination models.

Feature selection methods	Logistic regression		SVM		Random forest		GBM		Naive Bayes	
	training	validation	training	validation	training	validation	training	validation	training	validation
MI	0.79	0.73	0.76	0.71	0.72	0.70	0.76	0.74	0.74	0.60
L1- regularization	0.78	0.70	0.76	0.70	0.78	0.60	0.77	0.65	0.72	0.58
Tree model	0.79	0.61	0.78	0.69	0.77	0.67	0.78	0.73	0.79	0.70

SVM, support vector machine; GBM, gradient boosting machine; MI, mutual information.

**Figure 4:**
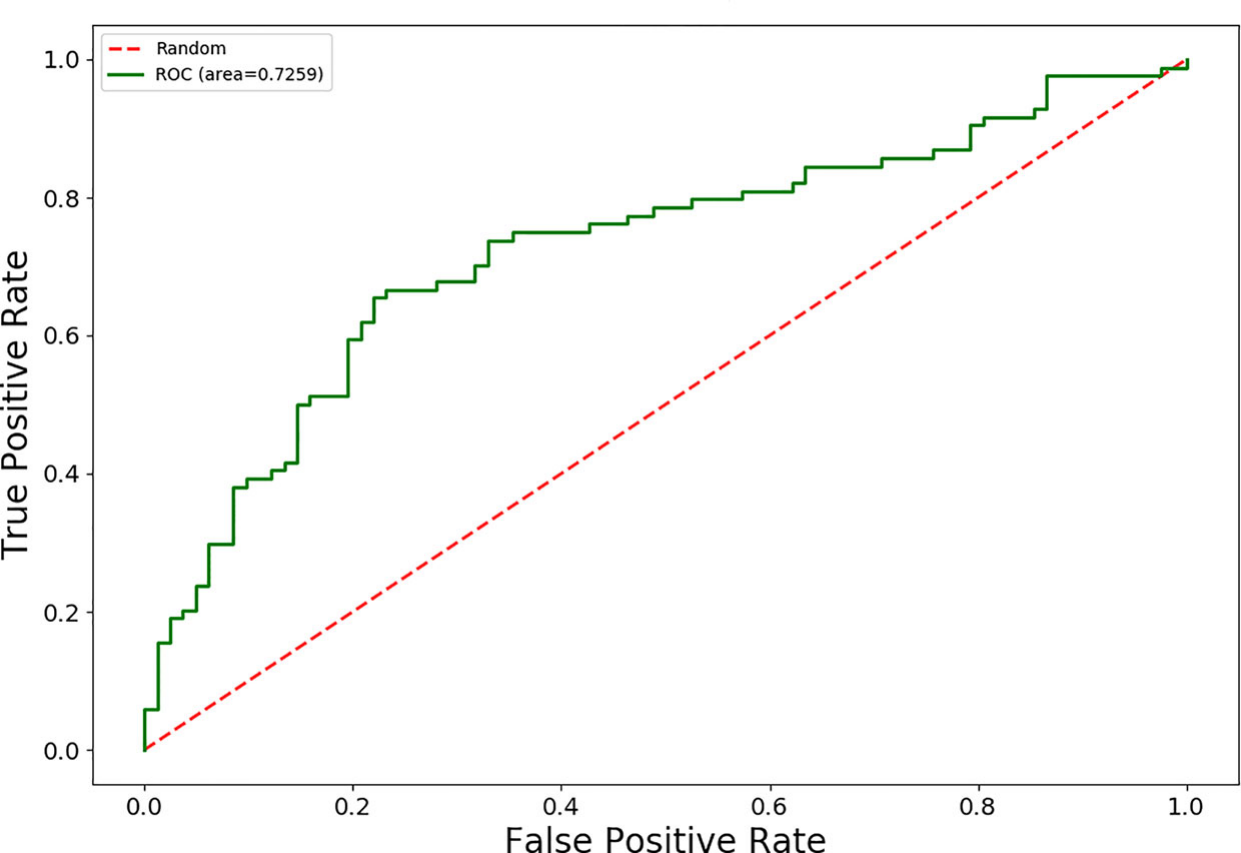
The ROC curves of the radiomics signature in the external validation set.

**Figure 5:**
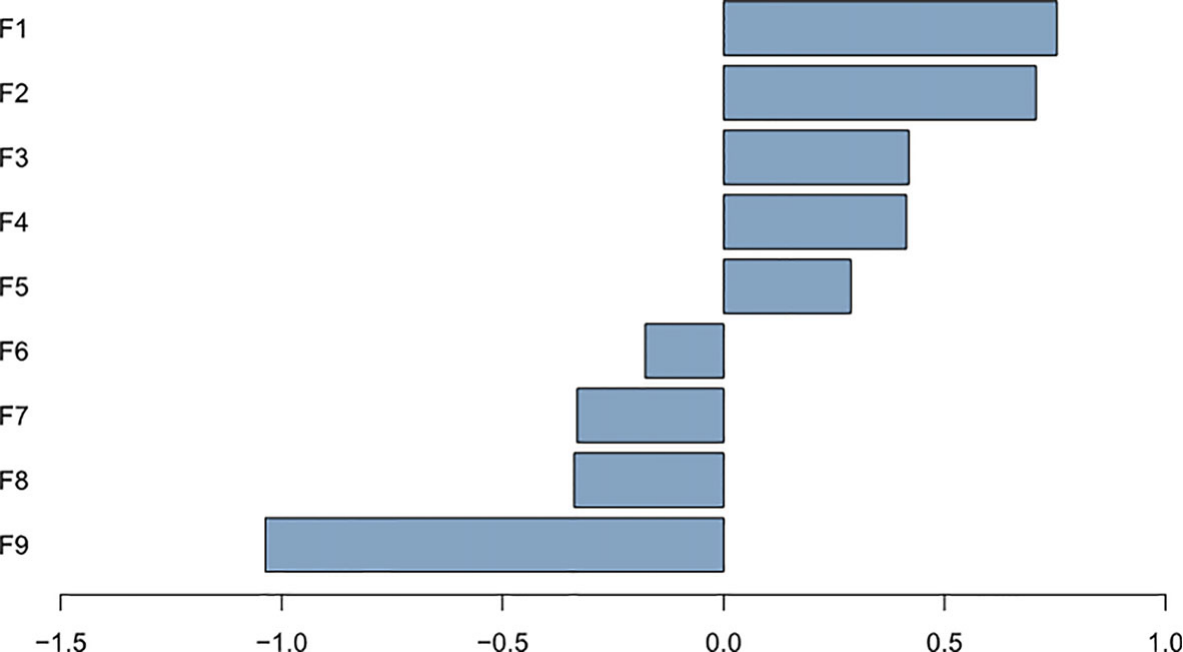
Plot of regression coefficients for features F1: energy; F2: location; F3: correlation; F4: coarseness; F5: gray level nonuniformity; F6: dissimilarity; F7: compactness; F8: cluster tendendcy; F9: number of voxels.

The nine most significant features included one clinical characteristic and eight radiomic features were identified after dimensionality reduction (**Figure 4**). The eight radiomic features included: energy, correlation, dissimilarity and difference entropy in gray level co-occurrence matrix(GLCM), coarseness in neighbor intensity difference roundness, gray level nonuniformity in gray level run length matrix(GLRLM), compactness and number of voxels in shape. Furthermore, one clinical feature was location (**Figure 5**).

## Discussion

We developed a predictive model combining CT image features and clinical characteristics to predict the MSI status in CRC patients preoperatively. The model was validated using an external independent dataset from multiple centers, and proved to have both predictive value and generalization ability. The model could be potentially used in the clinical setting.

Pathological examinations demonstrated that tumor heterogeneity was significantly different between MSI-H and MS-L/S CRC. Over 50% of MSI-H tumors exhibited two or more than two types of tissues, e.g., mucinous and solid, or glandular and mucinous. In contrast, only 11% of MS-L/S tumors had this phenomenon (17). MSI-H tumors have higher vascular density than MS-L/S tumors (18). Such differences in microstructure made it possible to stratify MSI status in CRC using the radiomics model. Radiomics mines high-throughput quantitative imaging features, which are then screened, clustered, analyzed, and modeled to recognize and predict tumor heterogeneity. Thus, the association between radiological features and MSI status in CRC could be obtained.

Moreover, clinical characteristics are associated with a tumor’s molecular subtype. In this study, the age and tumor location differed significantly between MSI-H and MS-L/S groups. However, after screening for features, only tumor location remained significant, and was eventually used to construct the prediction model. The results were consistent with the fact that MSI-H CRC tumors are mostly located in the right-side colon (19, 20). The studies showed that the proximal and distal colon have distinct embryonic origins and are exposed to different environmental carcinogens, which lead to different biological properties (such as MSI status) (21) and consequently affect the tumor’s responses to chemotherapeutics and targeted drugs.

The radiomics-based preoperative evaluation of CRC is mainly focused on pre-surgical staging, lymph nodes status, and prediction of molecular subtypes. Among the numerous molecular subtypes of CRC, MSI status is of great significance to guide clinical management (22). First, MSI status is a potential screening tool for hereditary non-polyposis colorectal cancer (HNPCC). Second, MSI status is a prognostic biomarker, with MSI-H indicating better outcomes in CRC stage II patients compared with MS-L/S (23). Patients with MSI-H CRC stage II had a lower risk for cancer-related death. MSI status also predicts the local invasion ability of the tumor (24, 25). Third, MSI status can potentially predict chemotherapy response. Cox proportional hazard analysis had demonstrated that MSI-H and CpG island methylation phenotype status are the most important prognostic factors for poor outcomes in patients treated with 5-FU therapy (26). Patients with MSI-H CRC stage II and III will not benefit from 5-FU therapy (27). Fourth, the TNM staging system, which only takes into account the current tumor condition, cannot predict the invasiveness of the tumor or the effectiveness of defensive immune responses. Some subtypes of CRC, despite having a lower TNM grade, can have a worse clinical presentation. Therefore, a more detailed molecular subtype classification of CRC is necessary. Radiomics-based prediction of MSI status could complement the TNM staging system, and provide additional prognostic value in the clinical setting (19).

Currently, there were few studies about using a contrast-enhanced CT based radiomics model to predict MSI status in CRC patients preoperatively (14, 15, 28). The AUC values obtained by Fang et al. (14) and Pernicka et al. (15) both based on CT images, were 0.75 and 0.80, respectively, and the AUC values of our training set were not significantly different from theirs, whereas the AUC value obtained in the study by Wu et al. (28) was 0.961, which was extremely high. However, their study was based on the iodine-based material decomposition images which were different from our method. These studies demonstrated that radiomics analysis had some value for predicting MSI status in CRC preoperatively. However, all the studies had smaller sample sizes, a single center, and were not validated by an independent dataset. This is not an uncommon problem in radiomics research. In the different platforms and studies, the radiomics analysis process varies vastly regarding the imaging equipment, image acquisition parameters, pre-processing of the radiological images, ROI segmentation methods, and feature extractions. Moreover, there are kinds of radiomics analysis softwares, the radiomics features are not standardized and normalized among different research groups. These factors pose a significant challenge to the comparison of radiomics results, and consequently affect the accountability of radiomics studies (29). A multi-center study can provide more diversified radiological data, better elucidate tumor heterogeneity, follow the trend of precision medicine, and lay a solid foundation for the clinical application translation of the radiomics model to predict MSI status in CRC. Our study is advantageous because it is a multi-center study. We used data from the largest institution as a training dataset, while the data from the other three institutions was used as a validation dataset. The results indicated that our model had good generalization ability.

Computed tomography is recommended by the NCCN guidelines as the preferred imaging examination for colorectal cancer in clinical practice. Using the radiomics model based on CT images to predict MSI status preoperatively offers several advantages, as it is non-invasive, objective, and easy to use. For the patients with high-risk tumors who need neoadjuvant chemotherapy prior to surgery, CT-imaging based radiomics model has the potential for early identification of MSI status without invasive histologic testing. Meanwhile, it is expected to be an image biomarker for predicting poor outcomes in CRC patients treated with 5-FU therapy.

This study has some limitations. First, this was a retrospective study, which inherently has selection bias. Second, we only considered the radiomic features extracted from the portal phase of enhancement. The regular CT images and the arterial phase of contrast enhancement were not analyzed. Third, because the CRC tumors had irregular shapes, we had to draw the ROIs manually, which inevitably caused some variations in tumor contouring. Fourth, the software IBEX we used may not be compatible with IBSI. In the future research, we will try to use pyradiomics instead of IBEX. Fifth, although we used patients from four institutions, the sample size of this study was relatively small. Further investigation with a larger sample size will be required.

In summary, the prediction model that was developed using CT image features and clinical characteristics has potential to predict MSI status in patients with CRC. However, this model needs further validation with more samples before it can be translated into clinical application. The results of this study lay the foundation for further radiomics studies in CRC.

## Data Availability Statement

The original contributions presented in the study are included in the article/supplementary material. Further inquiries can be directed to the corresponding author.

## Ethics Statement

The studies involving human participants were reviewed and approved by Institutional Review Board of the First Affiliated Hospital of Zhejiang University School of Medicine, the First Affiliated Hospital of Wannan Medical College, Cancer Hospital of the University of Chinese Academy of Sciences and Hangzhou Hospital of Traditional Chinese Medicine. The ethics committee waived the requirement of written informed consent for participation.

## Author Contributions

Study design, data analysis, manuscript writing, and manuscript approval were performed by ZL, and ZF, they are accountable for all aspects of the work. CT data collection, analysis and manuscript approval were performed by QZ, LZ, and MW. Pathological data analysis and interpretation, statistical analysis, and manuscript approval were performed by FY. Study design and manuscript approval were performed by WX and FC. Statistical analysis and manuscript approval were performed by CH. All authors contributed to the article and approved the submitted version.

## Conflict of Interest

CH was employed by the company Beijing Deepwise & League of PHD Technology Co, Ltd.

The remaining authors declare that the research was conducted in the absence of any commercial or financial relationships that could be construed as a potential conflict of interest.

## Abbreviations

CRC, Colorectal cancer; MSI, Microsatellite instability; MMR, Mismatch repair; MSI-H, MSI high-frequency; MSI-L, MSI low-frequency; MSS, MSI-stability; ESMO, European Society of Medical Oncology; IHC, Immunohistochemistry; ROI, Region of interest; mRMR, minimal Redundancy Maximal Relevance; AUC, Area under the curve; GLCM, Gray level co-occurrence matrix; GLRLM, Gray level run length matrix; HNPCC, Hereditary non-polyposis colorectal cancer; SVM, Support vector machine; GBM, Gradient boosting machine; MI, Mutual information; BS, Brier score.
